# DNASE1L3 as a Novel Diagnostic and Prognostic Biomarker for Lung Adenocarcinoma Based on Data Mining

**DOI:** 10.3389/fgene.2021.699242

**Published:** 2021-11-15

**Authors:** Jianlin Chen, Junping Ding, Wenjie Huang, Lin Sun, Jinping Chen, Yangyang Liu, Qianmei Zhan, Gan Gao, Xiaoling He, Guowen Qiu, Peiying Long, Lishu Wei, Zhenni Lu, Yifan Sun

**Affiliations:** ^1^ Departments of Clinical Laboratory of Affiliated Liutie Central Hospital of Guangxi Medical University, Liuzhou, China; ^2^ Departments of General surgery of Affiliated Liutie Central Hospital of Guangxi Medical University, Liuzhou, China; ^3^ Departments of Respiratory Medicine of Affiliated Liutie Central Hospital of Guangxi Medical University, Liuzhou, China; ^4^ Departments of Clinical Laboratory of Liuzhou Maternity and Child Healthcare Hospital, Liuzhou, China; ^5^ Department of Clinical Laboratory of People’s Hospital Rong an County, Liuzhou, China; ^6^ Departments of Orthopedics of Affiliated Liutie Central Hospital of Guangxi Medical University, Liuzhou, China

**Keywords:** LUAD, DNASE1L3, diagnostic, prognostic, TCGA database

## Abstract

Previous researches have highlighted that low-expressing deoxyribonuclease1-like 3 (DNASE1L3) may play a role as a potential prognostic biomarker in several cancers. However, the diagnosis and prognosis roles of DNASE1L3 gene in lung adenocarcinoma (LUAD) remain largely unknown. This research aimed to explore the diagnosis value, prognostic value, and potential oncogenic roles of DNASE1L3 in LUAD. We performed bioinformatics analysis on LUAD datasets downloaded from TCGA (The Cancer Genome Atlas) and GEO (Gene Expression Omnibus), and jointly analyzed with various online databases. We found that both the mRNA and protein levels of DNASE1L3 in patients with LUAD were noticeably lower than that in normal tissues. Low DNASE1L3 expression was significantly associated with higher pathological stages, T stages, and poor prognosis in LUAD cohorts. Multivariate analysis revealed that DNASE1L3 was an independent factor affecting overall survival (HR = 0.680, *p* = 0.027). Moreover, decreased DNASE1L3 showed strong diagnostic efficiency for LUAD. Results indicated that the mRNA level of DNASE1L3 was positively correlated with the infiltration of various immune cells, immune checkpoints in LUAD, especially with some m6A methylation regulators. In addition, enrichment function analysis revealed that the co-expressed genes may participate in the process of intercellular signal transduction and transmission. GSEA indicated that DNASE1L3 was positively related to G protein-coupled receptor ligand biding (NES = 1.738; P adjust = 0.044; FDR = 0.033) and G alpha (i) signaling events (NES = 1.635; P adjust = 0.044; FDR = 0.033). Our results demonstrated that decreased DNASE1L3 may serve as a novel diagnostic and prognostic biomarker associating with immune infiltrates in lung adenocarcinoma.

## Introduction

Lung cancer is the leading cause of cancer-related mortality worldwide. Lung adenocarcinoma (LUAD), also known as pulmonary adenocarcinoma, accounts for 60% of all lung cancers (Denisenko et al., 2018). LUAD mainly occurs in the distal lung and alveoli and is not easily reached by bronchoscopy, which poses a huge challenge in diagnosis and treatment of LUAD (Liu X. et al., 2021). However, activation of proto-oncogenes is known to play vital role in the formation of the vast majority of cancers, and gaining insight into its expression levels and disease-related prognosis may contribute to the development of effective diagnosis and prevention of lung adenocarcinoma.

Deoxyribonuclease 1-like 3 (DNASE1L3), a member of the deoxyribonuclease 1 family, is a DNASE1-like nuclease expressed in the endoplasmic reticulum. As one of the major serum nucleases (Rodriguez et al., 1997; Shiokawa and Tanuma, 2001), DNASE1L3 is predominantly produced by dendritic cells (DCs), macrophages (Mph), and neutrophils (NEU). It has been proved that DNASE1L3 plays vital role in DNA catabolism and cell apoptosis (Errami et al., 2012; Han et al., 2020). Increasing evidence has demonstrated that dysfunction of DNASE1L3 may cause immune responses against DNA and autoimmune diseases in mice and humans (Wilber et al., 2003; Al-Mayouf et al., 2011; Zochling et al., 2014; Sisirak et al., 2016; Sun et al., 2020). Study has also reported that DNASE1L3 was involved in breast cancer signal transduction (Sjoblom et al., 2006). Malecki et al. found that hyper expressive of DNASE1L3 gene can degrade the genome of ovarian cancer cells and lead to cell death (Malecki et al., 2013). In addition, investigators announced that the expression level of DNASE1L3 is closely align with the stage of clear cell renal cell carcinoma (Bhalla et al., 2017a). Recently, it has been reported that patients with high DNASE1L3 expression achieved significantly longer overall survival (OS) of HCC (Xu et al., 2019; Wang et al., 2020a). By the above, DNASE1L3 is highly likely to be contribute to the cancer genesis and progression. However, the role of DNASE1L3 in LUAD has not been well elucidated, which is the aim of our study.

In current study, we comprehensively analyzed DNASE1L3 expression data of LUAD patients in TCGA (The Cancer Genome Atlas), GEO (Gene Expression Omnibus), CPTAC dataset, and Human Protein Atlas (HPA) database. Using multi-omics analysis, we assessed the potential diagnosis or clinical prognosis efficiency of DNASE1L3 in lung adenocarcinoma. Moreover, we investigated the correlation of DNASE1L3 with tumor-infiltrating immune cells *via* R (version 3.6.3) GSVA package and ssGSEA (GSVA pack-built algorithm) package. Various bioinformatics tools were used to explore the genetic alteration of DNASE1L3, potential biological functions of DNASE1L3, the correlation with immune checkpoints, and m6A RNA methylation regulators of DNASE1L3 in LUAD. Our results confirmed that low DNASE1L3 expression might be a useful diagnostic and poor prognostic biomarker for lung adenocarcinoma.

## Data and Methods

### Expression and Transcription Analysis

The TIMER2.0 was employed to observe the expression difference of DNASE1L3 in 33 cancers of the TCGA project. A total of 526 lung adenocarcinoma patients and 57 normal including clinical data and RNA-seq of DNASE1L3 (*p* ≤ 0.05, |log2FC| ≥ 2) were downloaded from TCGA database by UCSC Xena website (https://xena.ucsc.edu/). Then, the gene expression of DNASE1L3 was analyzed basing on different clinical groups. The log2 [TPM (Transcripts per million) +1] or log2 [FPKM (Fragments Per Kilo base per Million) +1] transformed expression data was applied for data analysis. The CPTAC dataset (http://ualcan.path.uab.edu/analysis-prot.html) and Human Protein Atlas (HPA) database (http://proteinatlas.org) were utilized to compare protein level of DNASE1L3 in normal and LUAD tissues (Chen et al.). Two sets of LUAD chip datasets GSE40791 and GSE10072 were downloaded from the GEO (https://www.ncbi.nlm.nih.gov/geo/) database (Li et al., 2017) and utilized as validation sets to study the differential expression and diagnostic efficiency of DNASE1L3 in the study.

### Diagnostic and Survival Analysis

The diagnostic values of DNASE1L3 was calculated by pROC package (Robin et al., 2011) of R version 3.6.3, and the ROC curves were visualized by ggplot2 package (Wickham, 2016). The RNA-seq data in FPKM format were analyzed after log2 conversion. After that, LUAD cohort was clustered into low and high expression groups by median value of mRNA expression of DNASE1L3. The correlations between gene expression and OS, disease specific survival (DSS), and progress free interval (PFI) were analyzed *via* the Kaplan–Meier Plotter by R package (Liu et al., 2018). Then, the survival curves between subgroups (grouped by age, gender, pathological type, TNM stage, and smoking history) were plotted by the Kaplan–Meier analysis log-rank test. The HR with 95% CI was marked.

### Correlations Between DNASE1L3 Expression and Immune Characteristics

Data on immune cell infiltration were obtained from TCGA. R (version 3.6.3), GSVA package, and ssGSEA package were used for statistical analysis and visualization of the data (Hänzelmann et al., 2013). The relationship between the expression of DNASE1L3 and immune infiltration was analyzed (Liu et al., 2018). Spearman correlation of DNASE1L3 gene in lung adenocarcinoma was generated by correlation module, and its statistical significance was estimated. In addition, the relationship between DNASE1L3 expression and immune checkpoint marker expression levels by Spearman correlation analyses is shown.

### Tumor Mutation Profiles Analysis

Somatic mutation data were downloaded from the TCGA database and processed to identify the somatic variants and display somatic landscape by R package Maftools (Anand et al., 2018). The R package Somatic Signatures was utilize to describe the mutant signatures of lung adenocarcinoma samples (Gehring et al., 2015).

### Correlations Between DNASE1L3 Expression and m6A RNA Methylation Regulators

Differentially expressed m6A RNA methylation regulators (LUADs vs. normal tissues, high- and low-DNASE1L3 LUADs) were analyzed by the Mann–Whitney U test method in R (version R 3.6.3). *p* < 0.05 and Log2|FC| > 1 were used as the significance criteria. Subsequently, expression of m6A-related genes in 526 LUAD patients and 57 normal samples was visualized.

### Pathway and Enrichment Analysis

A total of 526 LUAD patients were separated into high- and low-DNASE1L3 expression groups according to DNASE1L3 median value. Limma package was used to identify differentially expressed genes (DEGs). The |log 2 (Fold Change) | larger than 1.5 and an adjusted *p*-value less than 0.05 were set as thresholds. The “pheatmap” and “EnhancedVolcano” R packages were employed to supply the heatmaps and volcano plots of the selected genes. In addition, we applied “ClusterProfiler” R package to conduct and visualize the Kyoto encyclopedia of genes and genomes (KEGG) and Gene Ontology (GO) analysis, including molecular function (MF), biological process (BP), and cell composition (CC), and adjusted *p* < 0.05 is considered to be enriched to a meaningful pathway (Yu et al., 2012). Further, we also used the R package “ClusterProfiler” to explore the Gene Set Enrichment Analysis (GSEA) of the DEGs in the two groups (Subramanian et al., 2005).

### Statistical Analysis

R software (version 3.6.3) was utilized for all statistical analyses. Mann–Whitney U test was used to compare the two groups. The Kruskal–Wallis test compares three or more differences group. Chi-square test was performed and corrected to pass Fisher’s exact test. *p* < 0.05 was considered statistically significant.

## Results

### Baseline Characteristics of Patients

A total of 526 LUAD patients containing the required clinical features were downloaded from the TCGA dataset in April 2021. Clinical information including age, gender, TNM stage, pathologic stage, residual tumor, DNASE1L3 expression, smoking history, overall survival, and disease-free survival are listed in Table 1. Among the 526 participants, 249 were male (46.5%) and 286 were female (53.5%). The residual tumor type of most patients was R0 (95.4%), and 93.5% of the patients at M0 stage. In terms of pathologic stage, 294 patients were stage I (55.8%), 123 patients were stage II (23.3%), 84 patients were stage III (15.9%), and 26 patients were stage IV (4.9%). Most patients were smokers (85.6%). The median age of all LUAD patients was 65 years.

**TABLE 1:** Clinical characteristics of the patients with lung adenocarcinoma.

Characteristic	Levels	Overall
Age, n (%)	≤65	255 (49.4%)
	>65	261 (50.6%)
Gender, n (%)	Female	286 (53.5%)
	Male	249 (46.5%)
T stage, n (%)	T1	175 (32.9%)
	T2	289 (54.3%)
	T3	49 (9.2%)
	T4	19 (3.6%)
N stage, n (%)	N0	348 (67.1%)
	N1	95 (18.3%)
	N2	74 (14.3%)
	N3	2 (0.4%)
M stage, n (%)	M0	361 (93.5%)
	M1	25 (6.5%)
Pathologic stage, n (%)	Stage I	294 (55.8%)
	Stage II	123 (23.3%)
	Stage III	84 (15.9%)
	Stage IV	26 (4.9%)
Residual tumor, n (%)	R0	355 (95.4%)
	R1	13 (3.5%)
	R2	4 (1.1%)
Smoking, n (%)	No	75 (14.4%)
	Yes	446 (85.6%)
DNASE1L3, n (%)	High	268 (50.1%)
	Low	267 (49.9%)
OS event, n (%)	Alive	343 (64.1%)
	Dead	192 (35.9%)
DSS event, n (%)	Alive	379 (76%)
	Dead	120 (24%)

Abbreviations: OS, overall survival; DSS, disease-free survival.

### Decreased Expression of DNASE1L3 in Lung Adenocarcinoma

**FIGURE 1:**
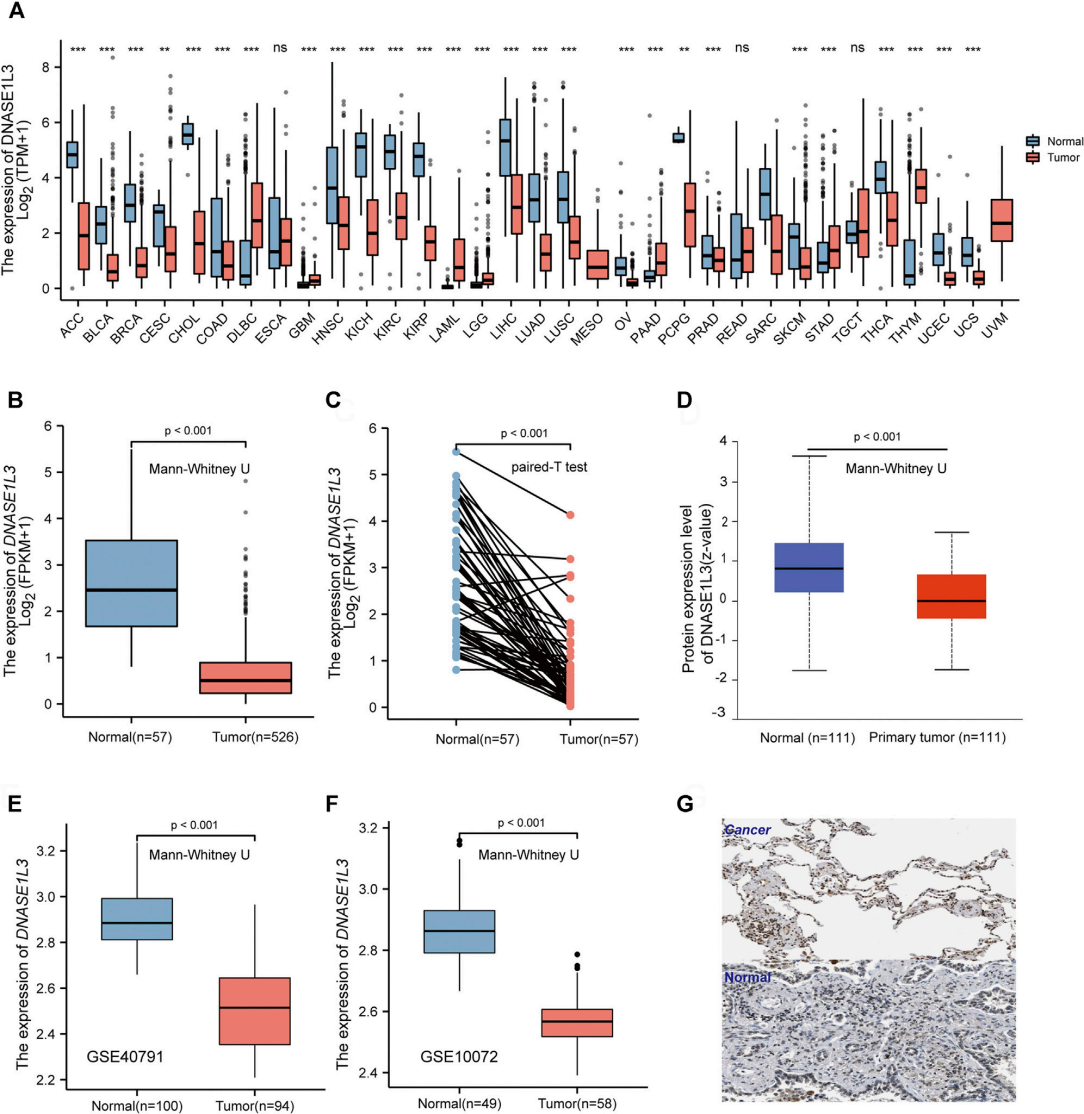
mRNA and protein expression level of DNASE1L3 in lung adenocarcinoma. **(A)** Human DNASE1L3 expression levels in different tumor types from TCGA database were determined by TIMER (^ns^ *p* > 0.05, **p < 0.01, ***p < 0.001). Based on the TCGA data, DNASE1L3 expression was significantly down-regulated in LUAD tissues compared to normal tissues **(B)** and noncancerous adjacent tissues (all *p* < 0.001) **(C)**. **(D)** Based on the CPTAC dataset, protein expression level of DNASE1L3 between normal tissue and primary tissue of LUAD (*p* < 0.001). **(E–F)** DNASE1L3 was expressed at a lower level in LUAD tissues than normal tissues (*p* < 0.05) from two different LUAD datasets. **(G)** Representative immunohistochemistry images of DNASE1L3 in LUAD and normal tissues derived from the HPA database.

To evaluate the DNASE1L3 mRNA levels in different tumors and normal tissues of multiple cancer types, we firstly examined DNASE1L3 expression using the RNA-seq data of multiple malignancies in TCGA. As shown in Figure 1A, DNASE1L3 expression shows significantly lower in the pancancers. Especially, the DNASE1L3 mRNA level was found to lowly expressed in LUAD comparing with normal tissue (Figure 1B, *p* < 0.001) and paired adjacent normal tissues (Figure 1C, *p* < 0.001). The differences in protein levels of DNASE1L3 were then analyzed by the CPTAC dataset and HPA database. As shown in Figure 1D, the protein expression level of DNASE1L3 was significantly down-regulated in LUAD tissues than in normal tissues (Figure 1D, *p* < 0.001). When showing the protein expression by immunohistochemistry images, the DNASE1L3 was still significantly down-regulated in LUAD tissues compared with normal samples (Figure 1G). To further verify the above conclusions, we further investigated DNASE1L3 expression in GSE40791 and GSE10072 datasets. We found that DNASE1L3 was indeed obviously expressed at a lower level in the LUAD tissues (Figures 1E,F, all *p* < 0.001). Taken together, all these results indicated that DNASE1L3 was remarkably down-regulated in LUAD patients at both mRNA and protein levels, implying a potential role in LUAD development and progression.

### Correlation of DNASE1L3 Expression With Clinical Features

**FIGURE 2:**
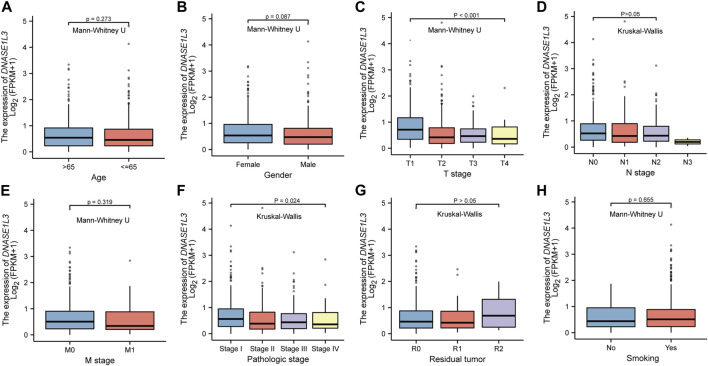
Box plot evaluating DNASE1L3 expression of patients with LUAD according to different clinical characteristics. **(A)** Age; **(B)** Gender; **(C)** T stage; **(D)** N stage; **(E)** M stage; **(F)** pathologic stage; **(G)** residual tumor; **(H)** smoking status.

Secondly, we explored the roles of DNASE1L3 in LUAD progression. The Mann–Whitney U test or Kruskal–Wallis rank sum test method was applied to analyze the correlation between the expression level of DNASE1L3 and age, gender, TNM stage, pathologic stage, residual tumor, and smoking status (Figures 2A–H). The results indicated that low expression of DNASE1L3 was significantly correlated with higher T stages (Figure 2C, *p* < 0.001) and pathologic stages (Figure 2F, *p* = 0.024). Moreover, as shown in Table 2. The expression of DNASE1L3 was associated with OS event (*p* < 0.001), pathologic stage (*p* = 0.013), and T stage (*p* < 0.001) but was not related to N stage (*p* = 0.214), M stage (*p* = 0.122), gender (*p* = 0.154), age (*p* = 0.253), residual tumor (*p* = 0.806), and smoking history (*p* = 0.762). Collectively, these results clarified that the low expression of DNASE1L3 gene was significantly correlated with advanced T stage and pathologic stage in LUAD.

**TABLE 2:** Relationship between the clinical features and DNASE1L3 expression in patients with lung adenocarcinoma.

Characteristic	Variable	Expression of DNASE1L3		p	χ^2^
		Low, n (%)	High, n (%)		
T stage	T1	60 (11.3%)	115 (21.6%)	< 0.001	25.15
	T2	166 (31.2%)	123 (23.1%)		
	T3	28 (5.3%)	21 (3.9%)		
	T4	11 (2.1%)	8 (1.5%)		
N stage	N0	166 (32%)	182 (35.1%)	0.214	4.48
	N1	53 (10.2%)	42 (8.1%)		
	N2	40 (7.7%)	34 (6.6%)		
	N3	2 (0.4%)	0 (0%)		
M stage	M0	180 (46.6%)	181 (46.9%)	0.122	2.4
	M1	17 (4.4%)	8 (2.1%)		
Pathologic stage	I	131 (24.9%)	163 (30.9%)	0.013	10.82
	II	70 (13.3%)	53 (10.1%)		
	III	47 (8.9%)	37 (7%)		
	IV	18 (3.4%)	8 (1.5%)		
Gender	Female	134 (25%)	152 (28.4%)	0.154	2.04
	Male	133 (24.9%)	116 (21.7%)		
Age	≤65	134 (26%)	121 (23.4%)	0.253	1.31
	>65	123 (23.8%)	138 (26.7%)		
Residual tumor	R0	186 (50%)	169 (45.4%)	0.806	0.43
	R1	8 (2.2%)	5 (1.3%)		
	R2	2 (0.5%)	2 (0.5%)		
Smoker	No	39 (7.5%)	36 (6.9%)	0.762	0.09
	Yes	220 (42.2%)	226 (43.4%)		
OS event	Alive	148 (27.7%)	195 (36.4%)	< 0.001	16.71
	Dead	119 (22.2%)	73 (13.6%)		

### Diagnostic Value of DNASE1L3 for Lung Adenocarcinoma

**FIGURE 3:**
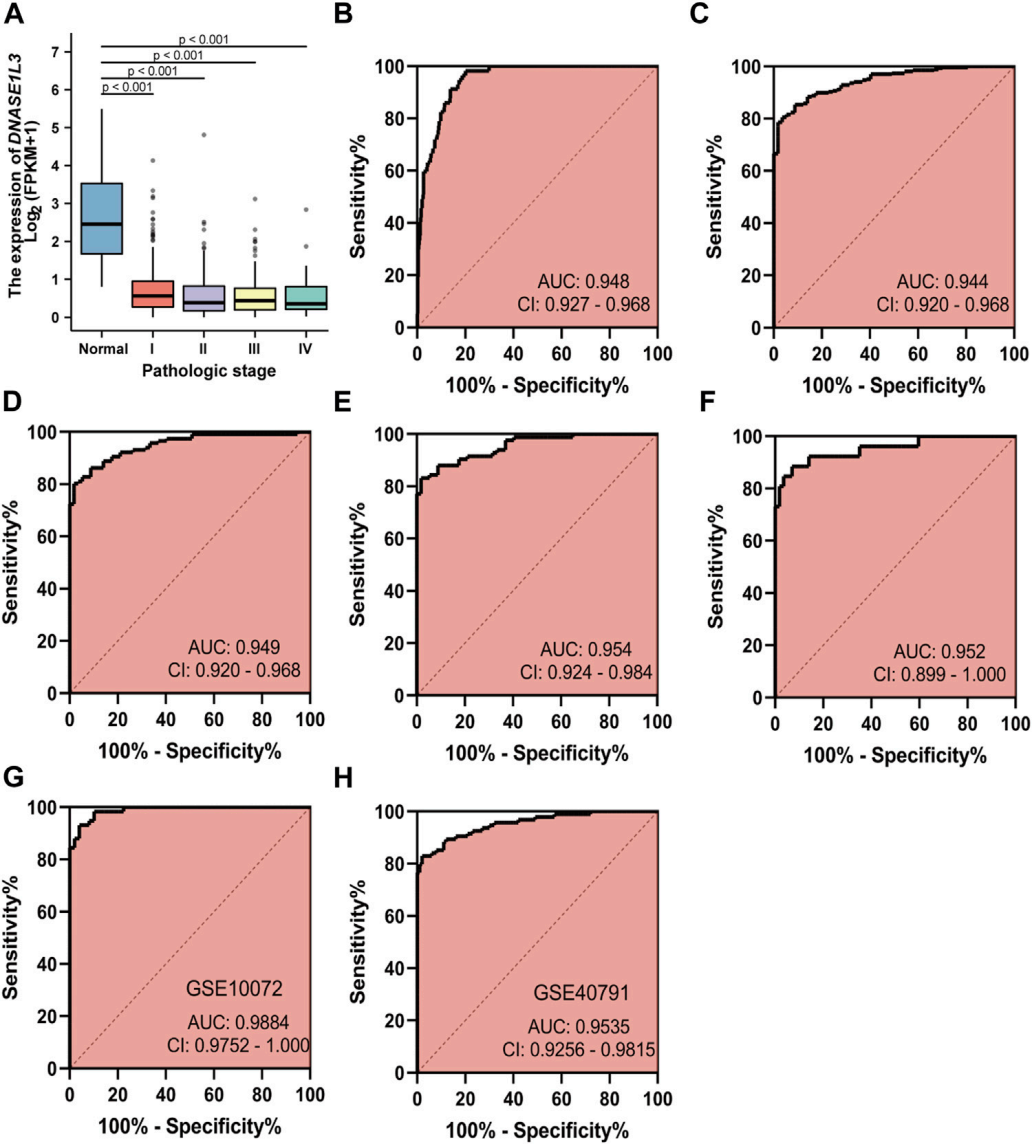
Diagnostic value of DNASE1L3 expression in patients with lung adenocarcinoma. The results of the TGCA dataset showed higher expression of DNASE1L3 in normal tissues than in pathologic stages of LUAD (all *p* < 0.001) **(A)**; ROC curves of DNASE1L3 expression in patients with LUAD, including normal vs. overall tumor **(B)**; normal vs. stage I tumor **(C)**; normal vs. stage II tumor **(D)**; normal vs. stage III tumor **(E)**; normal vs. stage IV tumor **(F)** based on the TGCA dataset; ROC curve of DNASE1L3 for LUAD through the GEO datasets **(G–H)**. AUC, area under the curve.

As decreased expression of DNASE1L3 had a trend to be associated with pathologic stage in LUAD patients, we hypothesized that it could be a better early diagnostic parameter for LUAD. Compared with the normal group, the expression of DNASE1L3 was significantly decreased at all pathological stages of LUAD (*p* < 0.0001, Figure 3A). The result showed that AUC value of DNASE1L3 for LUAD were 0.948 (95% CI, 0.927–0.968, sensitivity = 79.6% and specificity = 98.3%, Figure 3B). Then, the diagnosis at different pathological stages of LUAD was further analyzed. ROC analysis indicated that the AUC was calculated as 0.944 (strong) for stage I, 0.949 (strong) for stage II, 0.954 (strong) for stage III, and 0.952 (strong) for stage IV, respectively (Figures 3C–F). The diagnostic efficacy of DNASE1L3 for LUAD was also verified in GSE40791 and GSE10072 datasets. The AUC values for DNASE1L3 were 0.9984 (95% CI, 0.9752–1.000) and 0.9535 (95% CI, 0.9256–0.9815), respectively. These results indicated that DNASE1L3 could be invoked as a useful early diagnostic biomarker for lung adenocarcinoma.

### Prognostic Value of DNASE1L3 in Lung Adenocarcinoma

**FIGURE 4:**
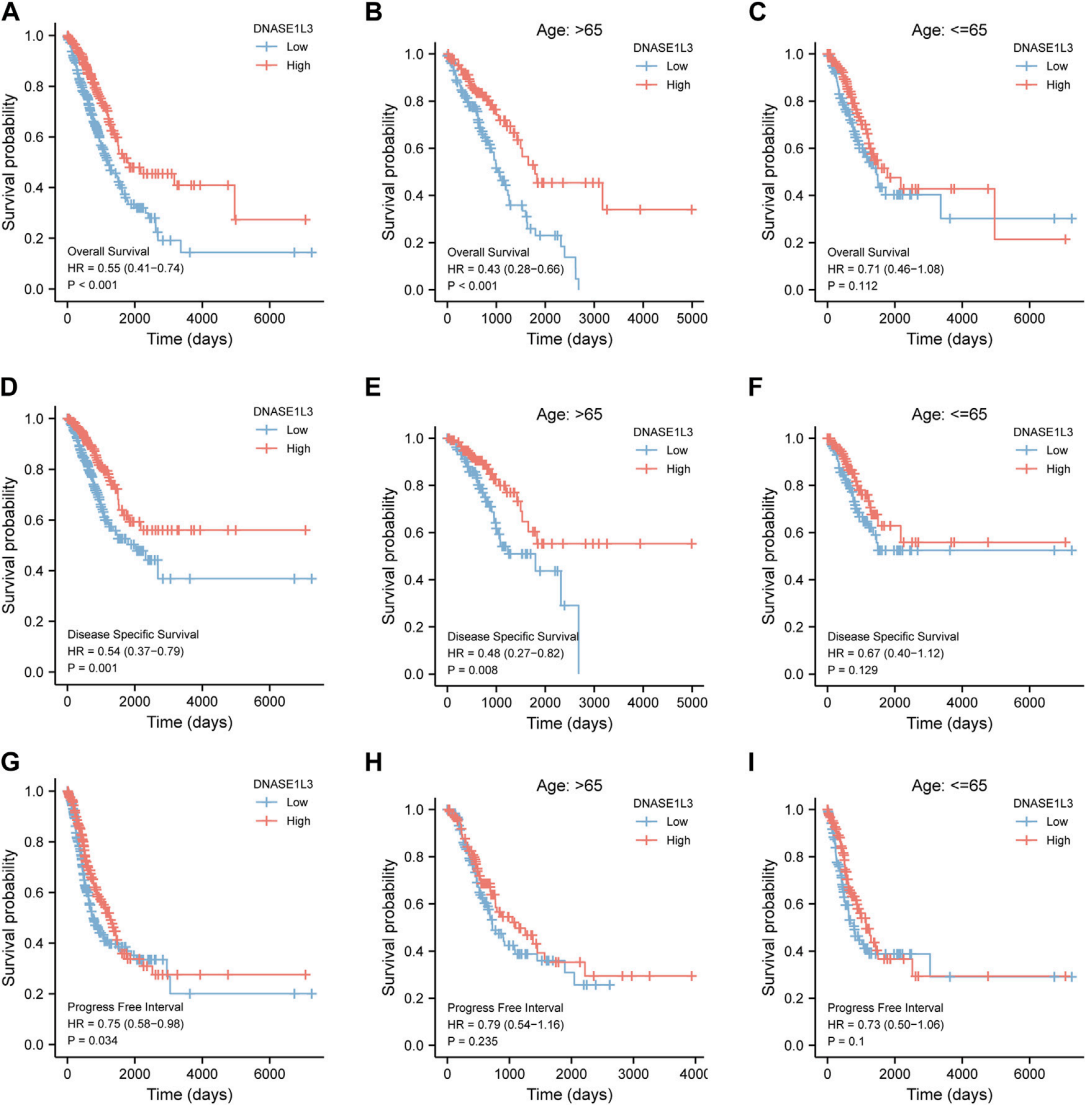
Survival analysis of DNASE1L3 in lung adenocarcinoma by OS, DSS, and PFI as well as stratified analysis by age. **(A–C)** Survival analysis of DNASE1L3 in lung adenocarcinoma by OS as well as stratified analysis by elderly patients and young patients, respectively. **(D–F)** Survival analysis of DNASE1L3 in lung adenocarcinoma by DSS as well as stratified analysis by elderly patients and young patients, respectively. **(G,H)** Survival analysis of DNASE1L3 in lung adenocarcinoma by PFI as well as stratified analysis by elderly patients and young patients, respectively. Notes: elderly patients: age > 65; young patients: age ≤ 65.

Next, we evaluated the prognostic values of DNASE1L3 in LUAD *via* the Kaplan–Meier plotter. Significant results were shown in Figure 4: lower mRNA expression of DNASE1L3 associated with poorer OS, DSS, and PFI in LUAD patients, respectively (Figure 4A HR = 0.55, 95% CI: 0.41–0.74, and *p* < 0.001; Figure 4D HR = 0.54, 95% CI: 0.37–0.79, and *p* = 0.001; Figure 4G HR = 0.75, 95% CI: 0.58–0.98, and *p* = 0.034). In addition, we performed stratified analyses of DNASE1L3 for OS, DSS, and PFI, respectively. The stratification was conducted by age, gender, T stage, N stage, M stage, pathological stage, and smoking status.

**FIGURE 5:**
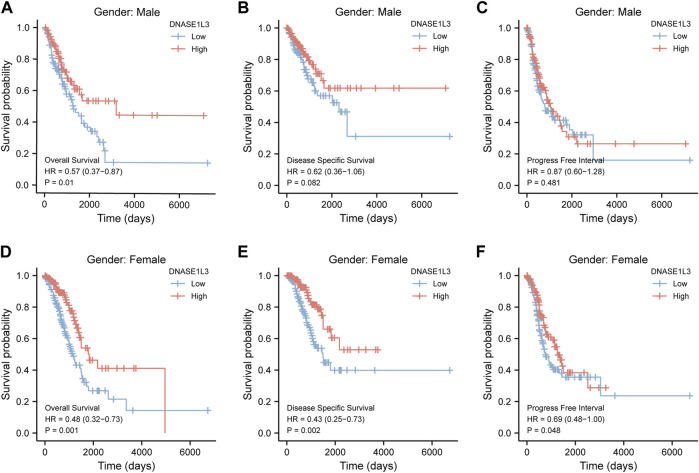
Stratified survival analysis of DNASE1L3 in lung adenocarcinoma by gender of OS, DSS, and PFI. **(A,D)** Stratified survival analysis of DNASE1L3 in lung adenocarcinoma of male and female of OS, respectively. **(B,E)** Stratified survival analysis of DNASE1L3 in lung adenocarcinoma of male and female of DSS, respectively. **(C,F)** Stratified survival analysis of DNASE1L3 in lung adenocarcinoma of male and female of PFI, respectively.

**FIGURE 6:**
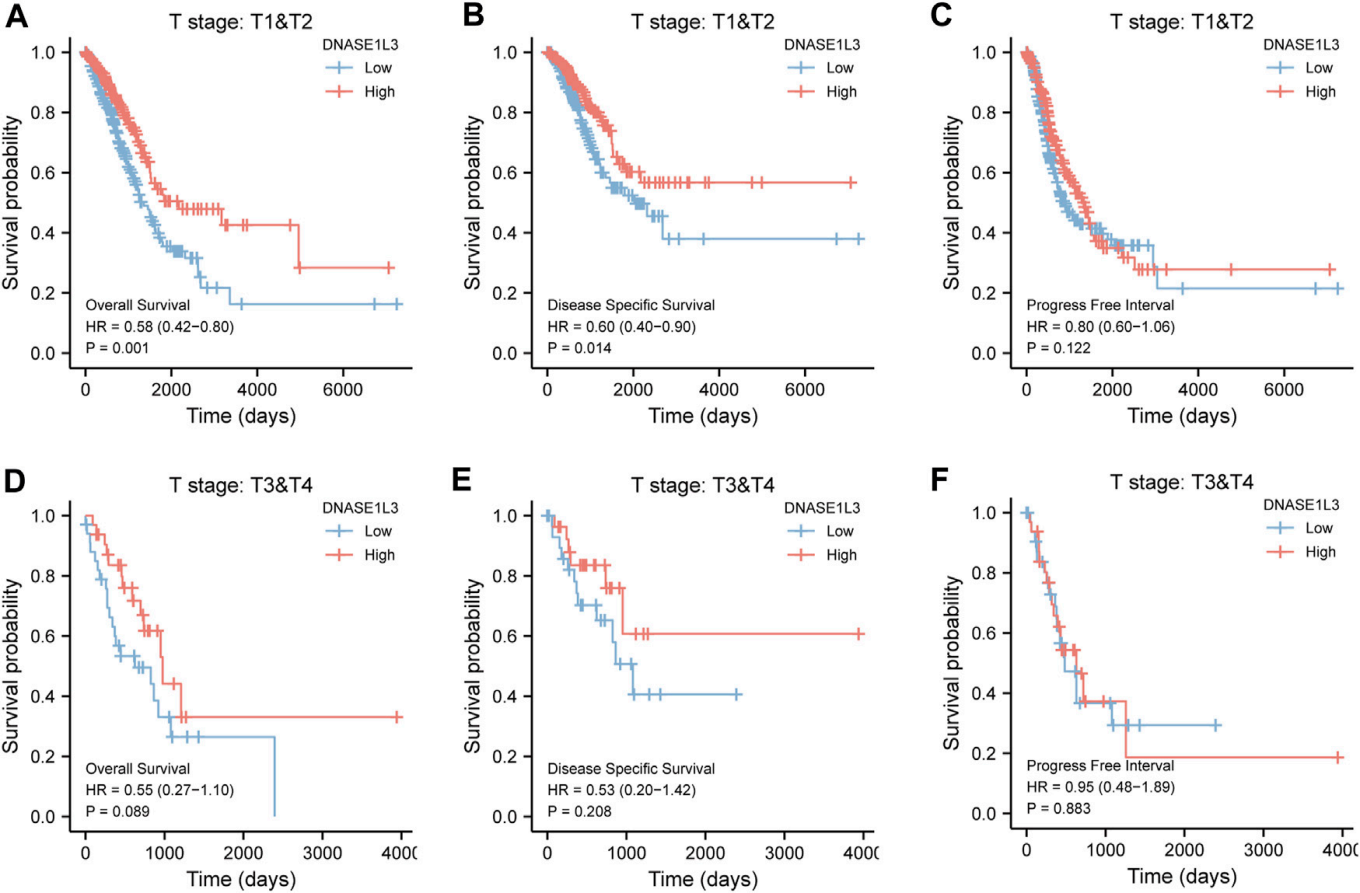
Stratified survival analysis of DNASE1L3 in lung adenocarcinoma by T stage of OS, DSS, and PFI. **(A,D)** Stratified survival analysis of DNASE1L3 in lung adenocarcinoma of T1&T2 and T3&T4 of OS, respectively. **(B,E)** Stratified survival analysis of DNASE1L3 in lung adenocarcinoma of T1&T2 and T3&T4 of DSS, respectively. **(C,F)** Stratified survival analysis of DNASE1L3 in lung adenocarcinoma of T1&T2 and T3&T4 of PFI, respectively.

**FIGURE 7:**
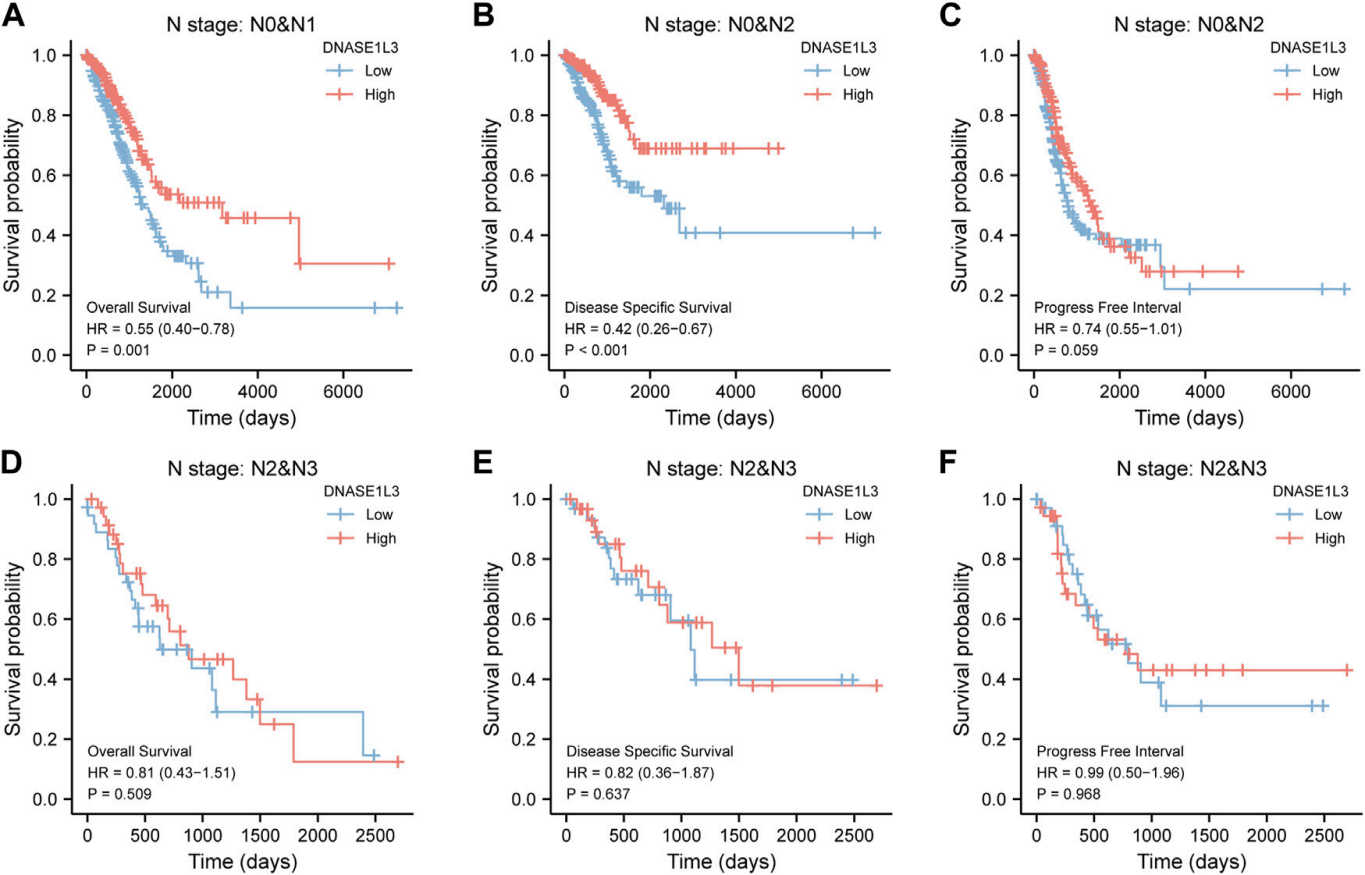
Stratified survival analysis of DNASE1L3 in lung adenocarcinoma by N stage of OS, DSS, and PFI. **(A,D)** Stratified survival analysis of DNASE1L3 in lung adenocarcinoma of N0&N1 and N2&N3 of OS, respectively. **(B,E)** Stratified survival analysis of DNASE1L3 in lung adenocarcinoma of N0&N1 and N2&N3 of DSS, respectively. **(C,F)** Stratified survival analysis of DNASE1L3 in lung adenocarcinoma of N0&N1 and N2&N3 of PFI, respectively.

**FIGURE 8:**
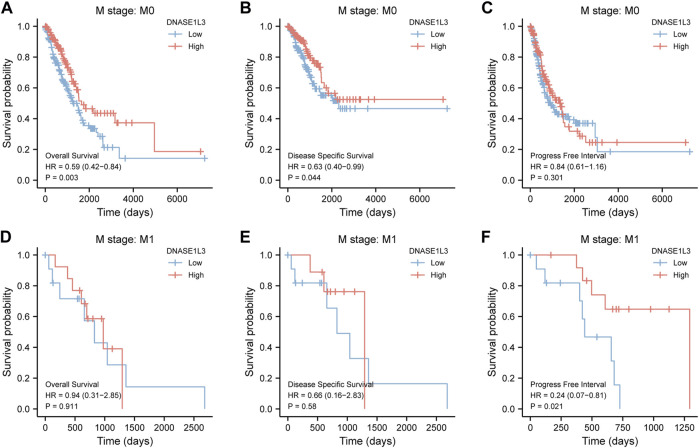
Stratified survival analysis of DNASE1L3 in lung adenocarcinoma by M stage of OS, DSS, and PFI. **(A,D)** Stratified survival analysis of DNASE1L3 in lung adenocarcinoma of M0 and M1 of OS, respectively. **(B,E)** Stratified survival analysis of DNASE1L3 in lung adenocarcinoma of M0 and M1 of DSS, respectively. **(C,F)** Stratified survival analysis of DNASE1L3 in lung adenocarcinoma of M0 and M1 of PFI, respectively.

**FIGURE 9:**
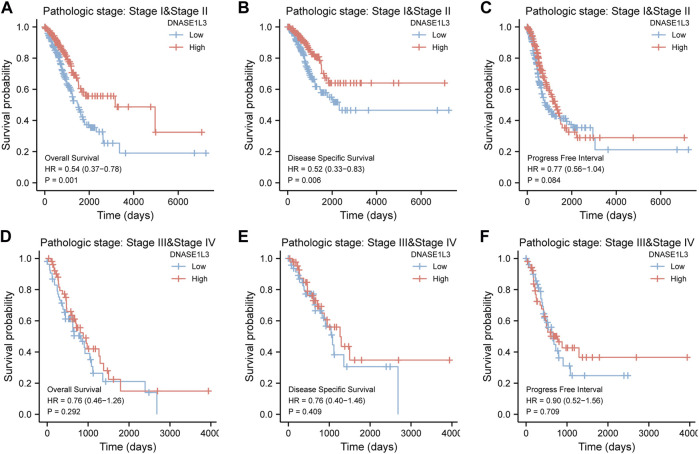
Stratified survival analysis of DNASE1L3 in lung adenocarcinoma by pathological stage of OS, DSS, and PFI. **(A,D)** Stratified survival analysis of DNASE1L3 in lung adenocarcinoma of early stage (Ⅰ &Ⅱ) and late stage (Ⅲ &Ⅳ) of OS, respectively. **(B,E)** Stratified survival analysis of DNASE1L3 in lung adenocarcinoma of early stage (Ⅰ &Ⅱ) and late stage (Ⅲ &Ⅳ) of DSS, respectively. **(C,F)** Stratified survival analysis of DNASE1L3 in lung adenocarcinoma of early stage (Ⅰ &Ⅱ) and late stage (Ⅲ &Ⅳ) of PFI, respectively.

**FIGURE 10:**
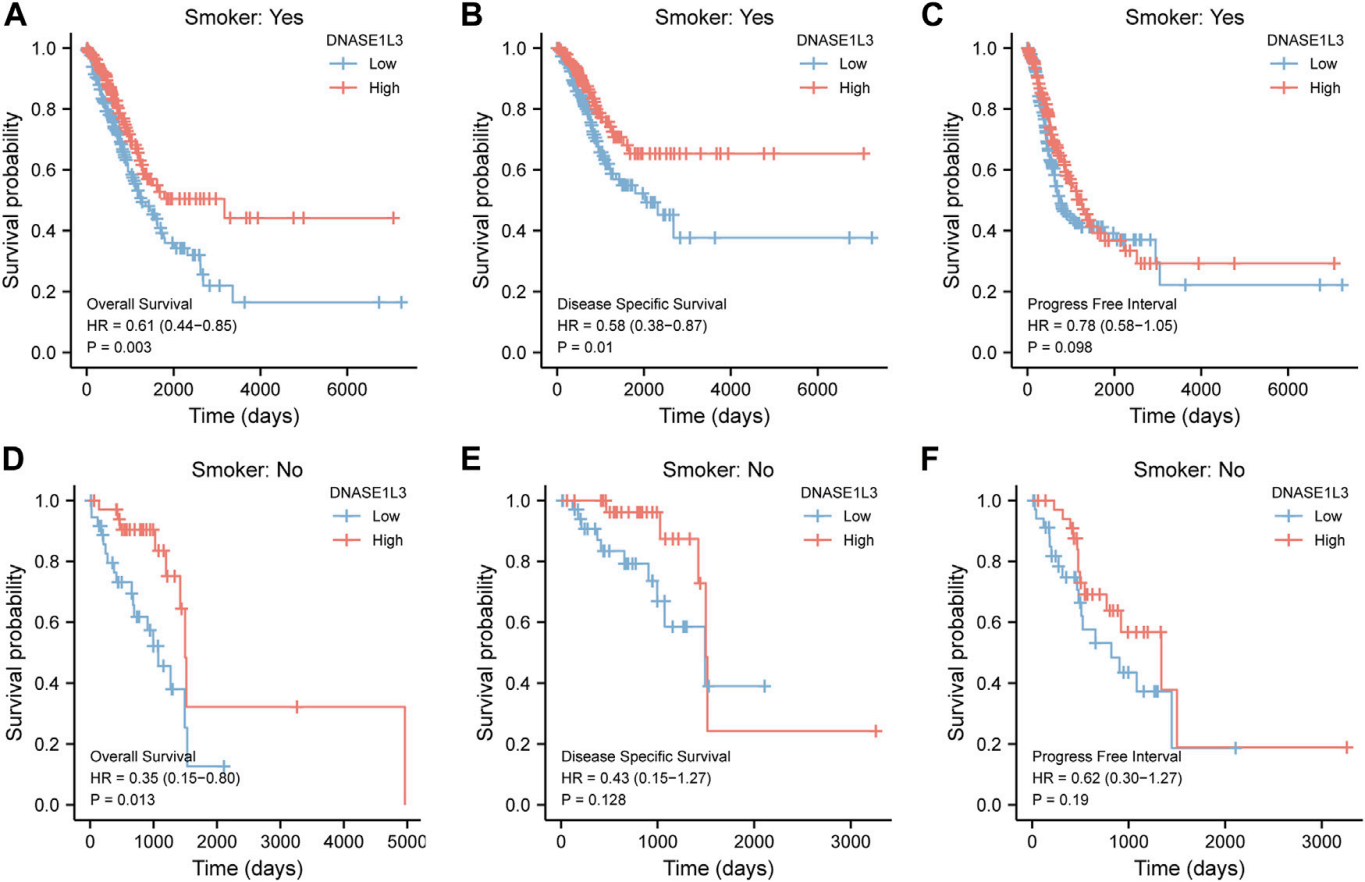
Stratified survival analysis of DNASE1L3 in lung adenocarcinoma by smoking status of OS, DSS, and PFI. **(A,D)** Stratified survival analysis of DNASE1L3 in lung adenocarcinoma of smoking and non-smoking of OS, respectively. **(B,E)** Stratified survival analysis of DNASE1L3 in lung adenocarcinoma of smoking and non-smoking of DSS, respectively. **(C,F)** Stratified survival analysis of DNASE1L3 in lung adenocarcinoma of smoking and non-smoking of PFI, respectively.

As shown in Figure 4, the OS and DSS of the low expression group of elderly patients (patients older than 65 years) was poorer than that of the high expression group (Figure 4B HR = 0.43 (0.28–0.66) *p* < 0.001; Figure 4E HR = 0.48 (0.27–0.82) *p* = 0.008). But there was no difference between the two groups in young patients (age ≤ 65) (Figures 4C,F). Also, as for PFI, there was no difference between the two groups of young patients or elderly patients (Figures 4H,I). Survival analysis stratified by gender is shown in Figure 5. As for male patients, only the OS of the low expression group was poorer than that of the high expression group (Figure 5A HR = 0.57 (0.37–0.87) *p* = 0.01), whereas the OS, DSS, and PFI of the low expression group of female patients were poorer than that of the high expression group, respectively (Figure 5D HR = 0.48 (0.32–0.73) *p* = 0.001; Figure 5E HR = 0.43 (0.25–0.73) *p* = 0.002; Figure 5F HR = 0.69 (0.48–1.00) *p* = 0.048). To more accurately assess survival outcomes, we performed a stratified survival score for T, N, and M stages. As shown in Figure 6, the OS and DSS of the low expression group of T1&T2 patients were poorer than that of the high expression group (Figure 6A HR = 0.58 (0.42–0.80) *p* = 0.001; Figure 6B HR = 0.60 (0.40–0.90) *p* = 0.014). However, there was no difference between the low expression group and high expression group of T3&T4 patients (Figures 6D,E). There was also no statistically significant difference in PFS between the two groups of T1&T2 and T3&T4 patients (Figures 6C,F). Similar results were obtained when analysis was stratified by N stage (N0&N1 vs. N2&N3) (Figures 7A–F). The results from stratified analysis by M stage were presented in Figure 8. The OS and DSS of the low expression group of M0 patients were poorer than that of the high expression group (Figure 8A HR = 0.59 (0.42–0.84) *p* = 0.003; Figure 8B HR = 0.63 (0.40–0.99) *p* = 0.044). But there was no difference between the two group of M1 patients (Figures 8D,E). There was also no statistically significant difference in PFS between the two groups of M0 patients (Figure 8C). However, the PFI of the low expression group of M1patients was poorer than that of the high expression group (Figure 8F HR = 0.24 (0.07–0.81) *p* = 0.021). Figure 9 shows results of the analysis stratified by pathological stage early stage (Ⅰ & Ⅱ) vs. late stage (Ⅲ &Ⅳ). The result demonstrated that analysis stratified by pathological stage (Figures 9A–F) showed similar results with T stage (T1&T2 vs. T3&T4) and N stage (N0&N1 vs. N2&N3). The results by stratified survival analysis of DNASE1L3 in lung adenocarcinoma by smoking status of OS, DSS, and PFI are shown in Figure 10. The OS and DSS of the low expression group of smoking patients was poorer than that of the high expression group [Figure 10A HR = 0.61 (0.44–0.8) *p* = 0.003; Figure 10B HR = 0.58 (0.38–0.87) *p* = 0.01]. In addition, the OS of the low expression group of non-smoking patients was poorer than that of the high expression group [Figure 10D HR = 0.35 (0.15–0.80) *p* = 0.013]. However, the PFI of the low expression group of smoking patients was not significant with the high expression group (Figure 10C), as well as the DSS and PFI of the two groups of non-smoking patients (Figures 10E,F).

**FIGURE 11:**
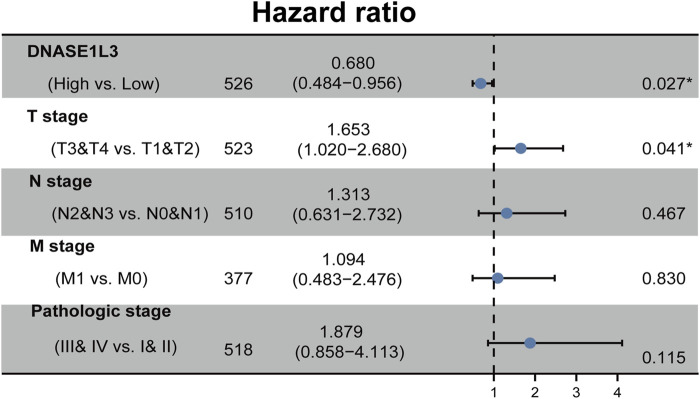
Forest plot of the multivariate Cox regression analysis in LUAD.

**FIGURE 12:**
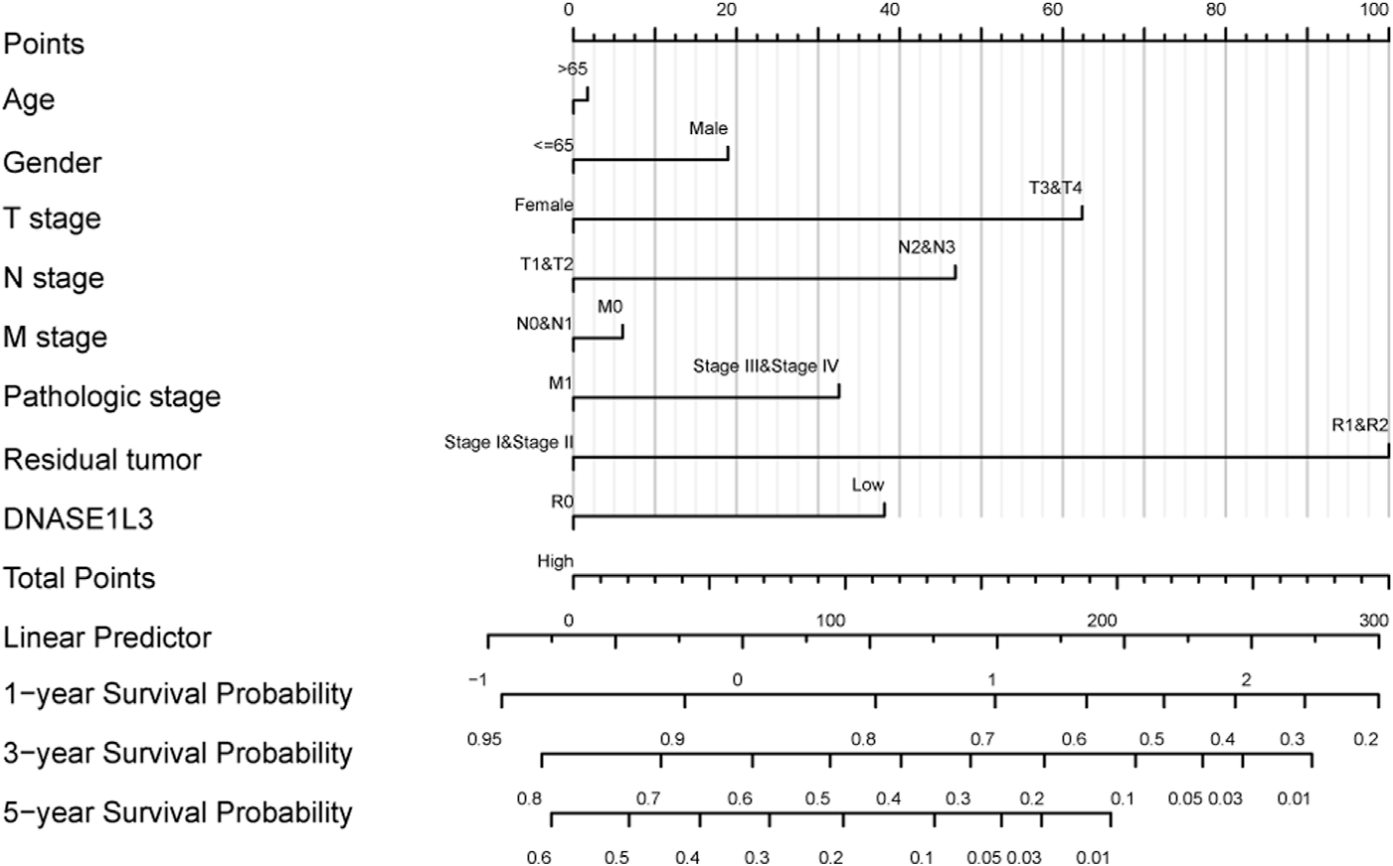
Nomogram for predicting probability of patients with 1-, 3-, and 5-years OS.

The prognostic significance of the DNASE1L3 level was further confirmed in a Cox regression analysis. In the univariate analysis, DNASE1L3 expression, T stage, N stage, M stage, and pathologic stage were all associated with the OS (Table 3, all *p* < 0.05). In the multivariate analysis, DNASE1L3 expression, along with T stage, was also associated with the OS, and the DNASE1L3 was an independent factor for the overall survival of LUAD patients (HR = 0.680; 95% CI: 0.484–0.956; *p* = 0.027, HR = 1.653; 95% CI: 1.020–2.680; *p* = 0.041) (Table 3 and Figure 11). Furthermore, according to the above results, a nomogram was performed to predict the1-, 3-, and 5-years survival probability of patients by combining the expression level of DNASE1L3 with clinical parameters (Figure 12). Taken together, the above results suggested that DNASE1L3 could function as a potential prognostic factor in LUAD.

**TABLE 3:** Univariate and multivariate analysis of overall survival in patients with lung adenocarcinoma.

Characteristics	Total (N)	Univariate analysis		Multivariate analysis	
		HR (95% CI)	*p* Value	Hazard ratio (95% CI)	*p* Value
DNASE1L3	526	0.550 (0.410–0.739)	**<0.001**	0.680 (0.484–0.956)	**0.027**
T stage	523	2.317 (1.591–3.375)	**<0.001**	1.653 (1.020–2.680)	**0.041**
N stage	510	2.321 (1.631–3.303)	**<0.001**	1.313 (0.631–2.732)	0.467
M stage	377	2.136 (1.248–3.653)	**0.006**	1.094 (0.483–2.476)	0.830
Pathologic stage	518	2.664 (1.960–3.621)	**<0.001**	1.879 (0.858–4.113)	0.115
Gender	526	1.070 (0.803–1.426)	0.642	—	—
Age	516	1.223 (0.916–1.635)	0.172	—	—
Smoker	512	0.894 (0.592–1.348)	0.591	—	—

Abbreviations: HR, hazard ratio; CI, confidence interval. Bold values indicate *p* < 0.05.

### DNASE1L3 Correlates With Immune Infiltration in Lung Adenocarcinoma

**FIGURE 13:**
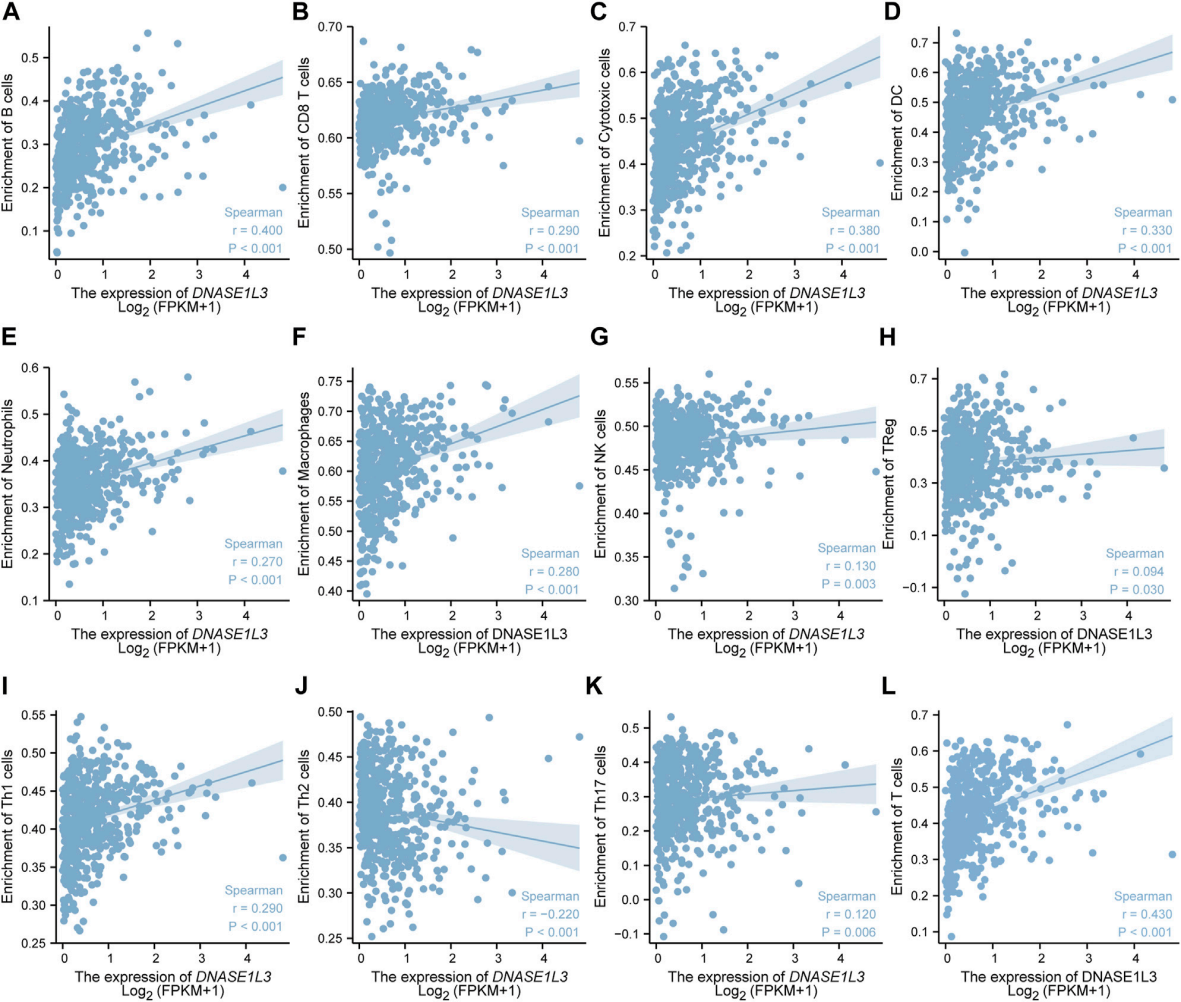
The relationship between DNASE1L3 expression and immune infiltration level in lung adenocarcinoma. The relationship between DNASE1L3 expression and B lymphocyte infiltration **(A)**, CD8 T cell infiltration **(B)**, cytotoxic cell infiltration **(C)**, DC infiltration **(D)**, neutrophil cell infiltration **(E)**, macrophage infiltration **(F)**, NK cell infiltration **(G)**, Treg cell infiltration **(H)**, Th1 cell infiltration **(I)**, Th2 cell infiltration **(J)**, Th17 cell infiltration **(K)**, and T cell infiltration **(I)**.

**FIGURE 14:**
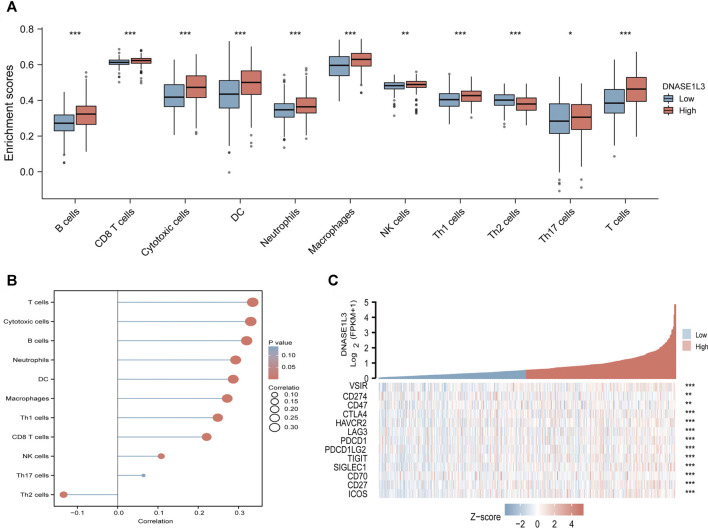
Immune characteristics of the TCGA-LUAD subtype. **(A)** Different infiltrating abundances of 11 immune cell types estimated by between high and low expression of DNASE1L3. **(B)** The relationship between DNASE1L3 expression and immune infiltration level. **(C)** Different expression of available emerging immunotherapeutic targets between high and low expression of DNASE1L3. *p < 0.05; **p < 0.01; ***p < 0.001.

The correlations between DNASE1L3 expression and immune infiltration were assessed with GSVA package and SSGSEA package in R. The Spearman rank correlation analysis revealed significant positive correlations between DNASE1L3 expression and CD8+T cells, cytotoxic cells, DCs, neutrophils, macrophages, NK cells, Treg cells, Th1 cells, Th17 cells, B cells, and T cells (all *p* < 0.05) (Figure 13, Figures 14A,B). However, DNASE1L3 indicated a negative correlation with Th2 cell in LUAD (Figure 13J, Figure 14B). To broaden our understanding of correlations between DNASE1L3 expression and immune infiltration, correlation analysis between DNASE1L3 and several immune checkpoints LUAD were performed. As shown in the Figure 14C, the gene expression levels of potential immune checkpoints, including CD47, CTLA4, VSIR, CD274, HAVCR2, LAG3, PDCD1, PDCD1LG2, TIGIT, SIGLEC1, CD70, CD27, and ICOS were positively correlated with the expression of DNASE1L3 (Figure 14C, all *p* < 0.05). It is highly likely that DNASE1L3 is involved in LUAD immune infiltration.

### Relationship Between DNASE1L3 Expression and Somatic Mutation Load

**FIGURE 15:**
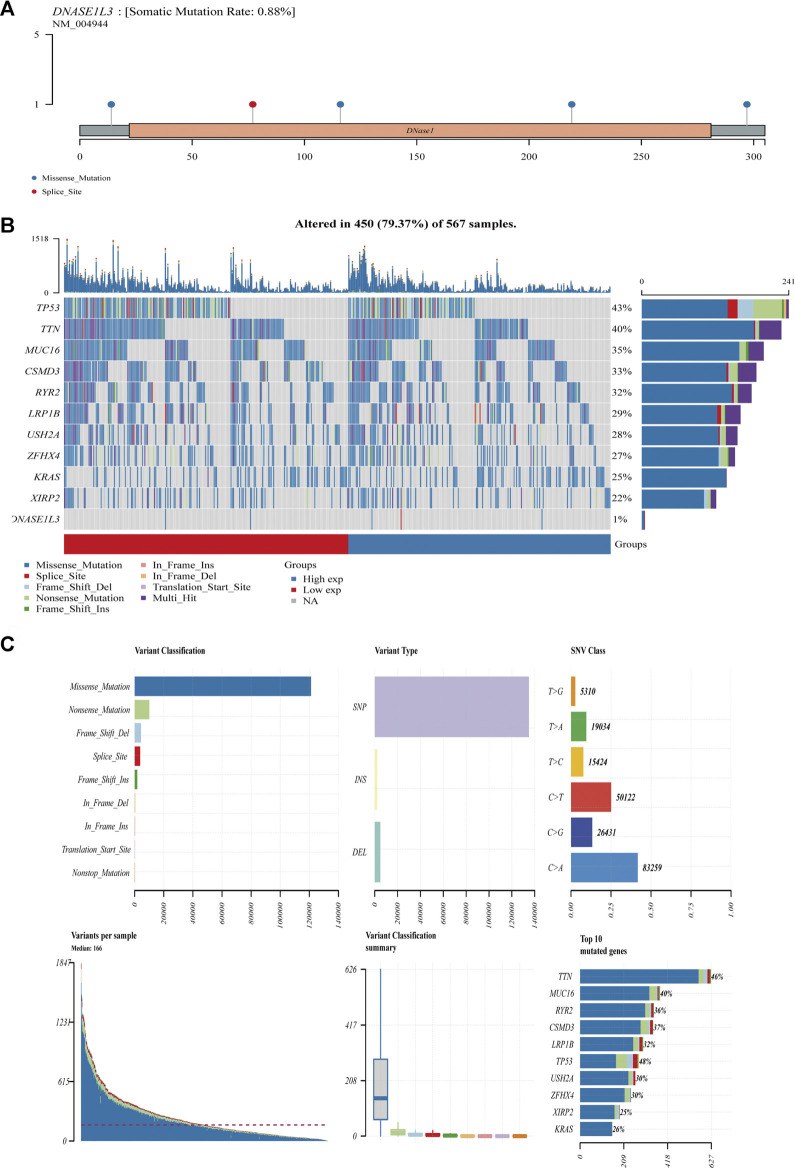
Mutation feature of DNASE1L3 in LUAD. The alteration frequency with mutation type and mutation site of DNASE1L3 **(A)**. Distribution of frequently mutated genes in different TCGA-LUAD subgroups. The upper bar plot shows the tumor mutation burden (TMB) for each patient, whereas the left bar plot indicates the gene mutation frequency in different groups **(B)**. The top 10 LUAD-correlated mutation genes in TCGA projects and the mutation frequency, variant classification, variant type, and SNV class of the mutated genes in high- and low-DNASE1L3 subgroups **(C)**.

We further analyzed the association between somatic mutation load and DNASE1L3 expression in 567 LUAD cases, which showed missense mutation as the predominant type of variant classification (Figures 15A,B). Meanwhile, detailed comparisons revealed that single-nucleotide variant (SNV) in LUADs occurred more frequently than insertion or deletion (Figure 15C). Moreover, it was shown that C > A and C > T were found to be the main SNV class in LUADs (Figure 15C). In addition, the top 10 mutated genes in LUADs with ranked percentages were calculated. The gene mutation status was also shown with a waterfall plot. Waterfall plots illustrate the mutation profiles of genes in LUADs with high/low-DNASE1L3, and the mutation types are represented using various colors at the bottom of the map (Figures). Boxplots were used to exhibit the mutation frequency of each gene in high- and low-DNASE1L3 subgroups (Figure 15B).

### Relationship Between DNASE1L3 Expression and m6A Regulatory Genes

Finally, we analyzed the expression of 20 m6A regulatory genes in LUADs (*n* = 535) and normal tissues (*n* = 59). Heat maps showed that 15 m6A-related genes were differentially expressed between LUADs and control tissues (Supplementary Figure S1A) and 9 m6A-related genes were differentially expressed between high- and low-DNASE1L3 expression of tumor samples (Supplementary Figure S1B). Specifically, the expression levels of IGF2BP1, IGF2BP3, VIRMA, HNRNPC, RBMX, YTHDF1, YTHDF2, METTL3, RBM15, and HNRNPA2B1were remarkably higher in LUADs than those in normal tissues (Supplementary Figure S1C, all *p* < 0.001).

### Enrichment Analysis of DNASE1L3-Related Partners

To further explore the potential biological function of DNASE1L3 gene in LUAD, enrichment analysist based on the DNASE1L3-relating differentially expressed genes (DEGs) was performed. According to Supplementary Figure S2A, 195 DEGs were obtained including 167 upregulated genes and 28 downregulated genes. Corresponding hierarchical clustering analysis of the top 5 up-regulated and down-regulated DNASE1L3 expression-correlated DEGs was displayed (Supplementary Figure S2B). Enrichment analyses including GO and KEGG analyses were applied to further determine the underlying molecular mechanisms of the DNASE1L3 in tumorigenesis. The results of GO analyses revealed that most of upregulated DEGs were linked to the events such as regulation of membrane potential, regulation of postsynaptic membrane potential, neurotransmitter receptor activity, and extracellular ligand-gated ion channel activity. Moreover, the results of KEGG pathway enrichment analysis of upregulated DEGs were mainly involved in neuroactive ligand-receptor interaction (Supplementary Figure S2C,D and Table 4). Furthermore, the GSEA showed DNASE1L3-associated DEGs significantly enriched in G protein-coupled receptor ligand biding (NES = 1.738; P adjust = 0.044; FDR = 0.033) and G alpha (i) signaling events (NES = 1.635; P adjust = 0.044; FDR = 0.033) (Supplementary Figure S2E,F). All these results demonstrated that DNASE1L3 might regulate the process of intracellular signal transduction and transmission, which could provide a new direction to the research on the crosstalk between tumor cells.

**TABLE 4:** Results of GO enrichment and KEGG pathway analysis.

Ontology	ID	Description	Gene ratio	*p* value	*p*.adjust	*q* value
BP	GO:0003341	Cilium movement	7/127	3.92e-07	7.81e-04	7.15e-04
BP	GO:0007586	Digestion	8/127	4.91e-06	0.005	0.004
BP	GO:0042391	Regulation of membrane potential	13/127	8.33e-06	0.006	0.005
BP	GO:0060078	Regulation of postsynaptic membrane potential	7/127	4.85e-05	0.016	0.015
BP	GO:0001976	Neurological system process involved in regulation of systemic arterial blood pressure	3/127	4.87e-05	0.016	0.015
CC	GO:0098889	Intrinsic component of presynaptic membrane	10/137	2.63e-10	6.19e-08	4.77e-08
CC	GO:0099240	Intrinsic component of synaptic membrane	12/137	1.66e-09	1.51e-07	1.16e-07
CC	GO:0099056	Integral component of presynaptic membrane	9/137	1.93e-09	1.51e-07	1.16e-07
CC	GO:0099699	Integral component of synaptic membrane	11/137	9.14e-09	5.37e-07	4.14e-07
CC	GO:0042734	Presynaptic membrane	10/137	1.88e-07	8.82e-06	6.79e-06
MF	GO:0030594	Neurotransmitter receptor activity	6/126	1.90e-04	0.033	0.029
MF	GO:0005230	Extracellular ligand-gated ion channel activity	5/126	1.96e-04	0.033	0.029
MF	GO:0099529	Neurotransmitter receptor activity involved in regulation of postsynaptic membrane potential	4/126	4.38e-04	0.038	0.033
MF	GO:0098960	Postsynaptic neurotransmitter receptor activity	4/126	5.09e-04	0.038	0.033
MF	GO:0015464	Acetylcholine receptor activity	3/126	5.62e-04	0.038	0.033
KEGG	hsa04080	Neuroactive ligand-receptor interaction	12/62	8.49e-06	9.09e-04	8.49e-04

Note: BP, biological process; CC, Cellular Component; MF: Molecular Function. P adjust value < 0.05 and q-value <0.25 were considered as significantly enriched.

## Discussion

DNASE1L3 can cleave both single and double stranded DNA, generating DNA fragments with 3-OH ends (Serpas et al., 2019) and involve in intranuclear DNA fragmentation during apoptosis and necrosis. DNASE1L3 gene expression and functions in carcinoma have been recently reported (Wang et al., 2020a; Liu J. et al., 2021; Deng et al., 2021). However, the significance of its expression in prognosis and diagnosis in patients with LUAD is largely unclear.

In this study, we firstly analyzed the DNASE1L3 gene expression profile in pan-cancer *via* TIMER20. Then, we analyzed transcriptional and protein expression levels of DNASE1L3 in LUAD. We also confirmed the expression of DNASE1L3 in LUAD through the GSE40791 and GSE10072 datasets. The results demonstrated that DNASE1L3 was indeed obviously expressed at a lower level in the LUAD tissues. DNASE1L3 gene expression and its potential prognostic impact on patients with LUAD have not been evaluated. Wang et al. (Wang et al., 2020a) reported that the expression level of DNASE1L3 was significantly decreased and associated with poor overall survival in hepatocellular carcinoma. Deng et al. (Deng et al., 2021) investigated the expression levels of DNASE1L3, and the results revealed the DNASE1L3 was a prognostic biomarker in cancer of the breast, kidney, liver, stomach, lung adenocarcinoma, and sarcoma *via* bioinformatics analysis using TCGA database. This is the first comprehensive study to evaluate DNASE1L3 gene expression in the prognosis of patients with LUAD. Analysis revealed that DNASE1L3 gene expression was negatively associated with OS event, pathologic, stage and T stage. Multivariate Cox analysis further confirmed that low DNASE1L3 expression was an independent prognostic factor for OS in patients with LUAD; T stage was associated with worse prognosis in LUAD, as suggested by the forest plot. Additionally, Kaplan–Meier plotter results suggested that the down-regulation of DNASE1L3 indicated poor prognosis of LUAD, which specifically reflected on the advanced clinical characteristics of tumor pathological stages and the depth of the primary tumor invasion. These results suggest that DNASE1L3 plays a tumor suppressive role in LUAD. In this paper, we noted that the M stage represented the state of distant metastasis, but had no effect on the expression of DNASE1L3. Combined with subsequent analysis, it was found that DNASE1L3 may affect tumor progression mainly by regulating the immune microenvironment, but may not promote distant metastasis of tumor in tumor tissues.

Gender is a key factor affecting individual cancer progression. There are significant gender differences in the incidence, aggressiveness, prognosis, and treatment response of various tumors. However, as shown in Figure 1C, there was no difference in the expression of DNASE1L3 between male and female cancer patients. As the body ages, humans suffer many diseases, including tumors. In this study, the patients were divided into two groups by age of 65; it was found that age was not related to the expression of DNASE1L3, nor was it direct factor affecting the prognosis of LUAD patients in the TCGA-LUAD dataset, as can be seen in Table 2. Smoking is the leading risk factor for lung cancer. However, in this paper, smoking or not is not related to the expression of DNASE1L3, and the specific reasons need to be further explored.

Tumor immune cell infiltration plays an important role in tumor prognosis and influences the response to immunotherapy (Zhang et al., 2016; Cavnar et al., 2017; Titov et al., 2021). Currently, several studies have demonstrated the importance of tumor-infiltrating immune cells and other immune molecules (including tumor-associated macrophages, natural killer cells, and dendritic cells) in the prognosis of lung adenocarcinoma (Liu W. et al., 2021; Mu et al., 2021; Shen et al., 2021). Therefore, we further analyzed the infiltration rates of various immune cells with the low and high expression levels of DNASE1L3 gene. We observed that the level of DNASE1L3 expression was positively linked to immune infiltration, especially for DCs, Mphs, and NEUs, which is similar to previous researches. In addition, in order to gain deeper insights into the immune landscape of LUAD, the expression of immune checkpoint molecules was investigated. By assessing the relationship between immune checkpoint molecule expression and DNASE1L3 profile, we found heterogeneity in the expression of immune checkpoint proteins in the immune microenvironment of LUAD. From the above results, we infer that the expression of DNASE1L3 involved in the immune infiltration may potentially impact the occurrence and development of lung adenocarcinoma.

As observed by Bhalla et al., DNASE1L3 was overexpressed in early stage of clear cell renal cancer and has some diagnostic ability to distinguish between early and late stage tumors (Bhalla et al., 2017a). However, its diagnostic significance in lung adenocarcinoma has not been reported. Our research first suggests and validates the diagnostic (strong) value of DNASE1L3 for lung adenocarcinoma.

Tumor mutation burden (TMB) is considered essential factors impacting on the occurrence and progression of tumor. Previously, Yue et al. (Yue et al., 2019) reported that a TMB relating gene has predictive accuracy for OS in LUAD patients. In our study, the mutation status of several cancer-related genes with high mutation probability in LUAD significantly affected the expression of DNASE1L3. The correlation between these mutated genes and the expression of DNASE1L3 suggests that differential expression of DNASE1L3 may play a regulatory role in LUAD. Nevertheless, further studies are needed to verify this hypothesis.

As a new dimension of gene expression control, RNA N6-methyladenosine (m6A) modification has attracted great academic interest in recent years. As the most abundant mRNA modification, m6A is involved in the regulation of the occurrence and development of tumors by controlling the expression of key genes, which has become the focus of research in recent years (Zhao et al., 2020). Previous studies have reported that abnormal m6A methylation modification may impact the development of lung cancer (Cheng et al., 2021). Our result showed that there are nine m6A-related regulators that were differentially expressed between high- and low-DNASE1L3 expression of LUAD. We speculate that the down expression of DNASE1L3 in lung adenocarcinoma is highly likely to be related to the abnormal regulation of m6A-related regulators; of course, the clear mechanism needs to be further studied.

In addition, we found that the up-expression phenotype of DNASE1L3 was associated with the events such as regulation of membrane potential, regulation of postsynaptic membrane potential, neurotransmitter receptor activity, and extracellular ligand-gated ion channel activity. Moreover, KEGG analysis revealed that DNASE1L3-associated DEGs were mainly involved in neuroactive ligand-receptor interaction. Furthermore, the GSEA showed significantly enriched in G protein-coupled receptor ligand biding and G alpha (i) signaling events. All these results indicated that DNASE1L3 might regulate the process of intracellular signal transduction and transmission, which could provide a new direction to the research on the crosstalk between tumor cells. However, such mechanisms require further investigation.

In a word, this is the first study to comprehensively analyze the relationship between DNASE1L3 expression level and early diagnosis and prognosis in patients with lung adenocarcinoma. Inevitably, this research has several limitations that need to be addressed. First, because the prognosis of DNASE1L3 in this research was based on data onto the TCGA datasets, additional clinical data are needed to verify it. Second, due to the small sample size, the effect of gene mutation and methylation of DNASE1L3 on prognosis cannot be carried out, and further supplement is needed. Thirdly, our study results are only limited to mRNA level, so it needs to be verified at protein level. In addition, there are still many questions to be solved, such as whether DNASE1L3 is related to chemotherapy resistance of LUAD, and how DNASE1L3 expression changes after chemotherapy? Meanwhile, what is the specific mechanism of DNASE1L3 in LUAD? Therefore, further molecular mechanism research is needed.

## Conclusion

In summary, this study provides novel evidences for the clinical and biological significance of DNASE1L3 in LUAD. Our results demonstrated that both the mRNA and protein levels of DNASE1L3 were noticeably downregulated in LUADs compared with normal tissues. The low expression level of DNASE1L3 was significantly associated with higher pathological stages, higher T stages, and poor prognosis in LUAD, and the DNASE1L3 expression level might be an independent prognostic factor of LUAD. Furthermore, from a series of bioinformatics analysis, we found mRNA level of DNASE1L3 was positively linked to the degree of infiltration of various tumor-infiltrating immune cells, the gene expression levels of potential immune checkpoint molecules, and some m6A methylation regulators in LUAD. Finally, DNASE1L3 showed strong early diagnostic value for LUAD. We conclude that the low expression level of DNASE1L3 may serve as a clinically useful diagnostic and prognostic biomarker, and potentially as a therapeutic target in LUAD.

## Data Availability

Publicly available datasets were analyzed in this study. This data can be found here: https://www.cancer.gov/about-nci/organization/ccg/research/structural-genomics/tcga.

## Supplementary Material

Supplementary Figure S1 Expression of m6A modification regulators in LUAD. (A) The heat map visualizes the expression levels of m6A RNA modification regulators in each tumor samples and normal control samples. (B) The expression levels of m6A RNA modification regulators in each high- and low-DNASE1L3 expression of tumor samples. (C) The box figure shows the differentially m6A RNA modification regulators in LUAD. The significance of the two groups of samples passed the Mann-Whitney U test. **p* < 0.05, ***p* < 0.01, ***p* < 0.001. **Abbreviations:** m6A: N6-methyladenosine.

Supplementary Figure S2 Enrichment analysis of DNASE1L3 expression-correlated DEGs in LUAD. (A) Volcano plot of DEGs between samples with high DNASE1L3 expression and low DNASE1L3 expression. (B) Heat maps of the top 5 up-regulated and down-regulated DNASE1L3 expression-correlated DEGs. (C) KEGG enrichment and GO enrichment analysis by DNASE1L3 expression-correlated upregulated DEGs. (D) Interaction networks of enriched biological processes. (E-F) Functional annotation of DEGs in LUAD patients with distinct DNASE1L3 levels. NES: normalized enrichment score; FDR: false discovery rate. FDR<0.25 and P adjust<0.05 is generally considered as significant enrichment.
