# Supplementary figures and images for: DNASE1L3 as a Novel Diagnostic and Prognostic Biomarker for Lung Adenocarcinoma Based on Data Mining
Source: Front Genet. 2021 Nov 15;12:699242. doi: 10.3389/fgene.2021.699242 (PMC8636112; doi:10.3389/fgene.2021.699242)

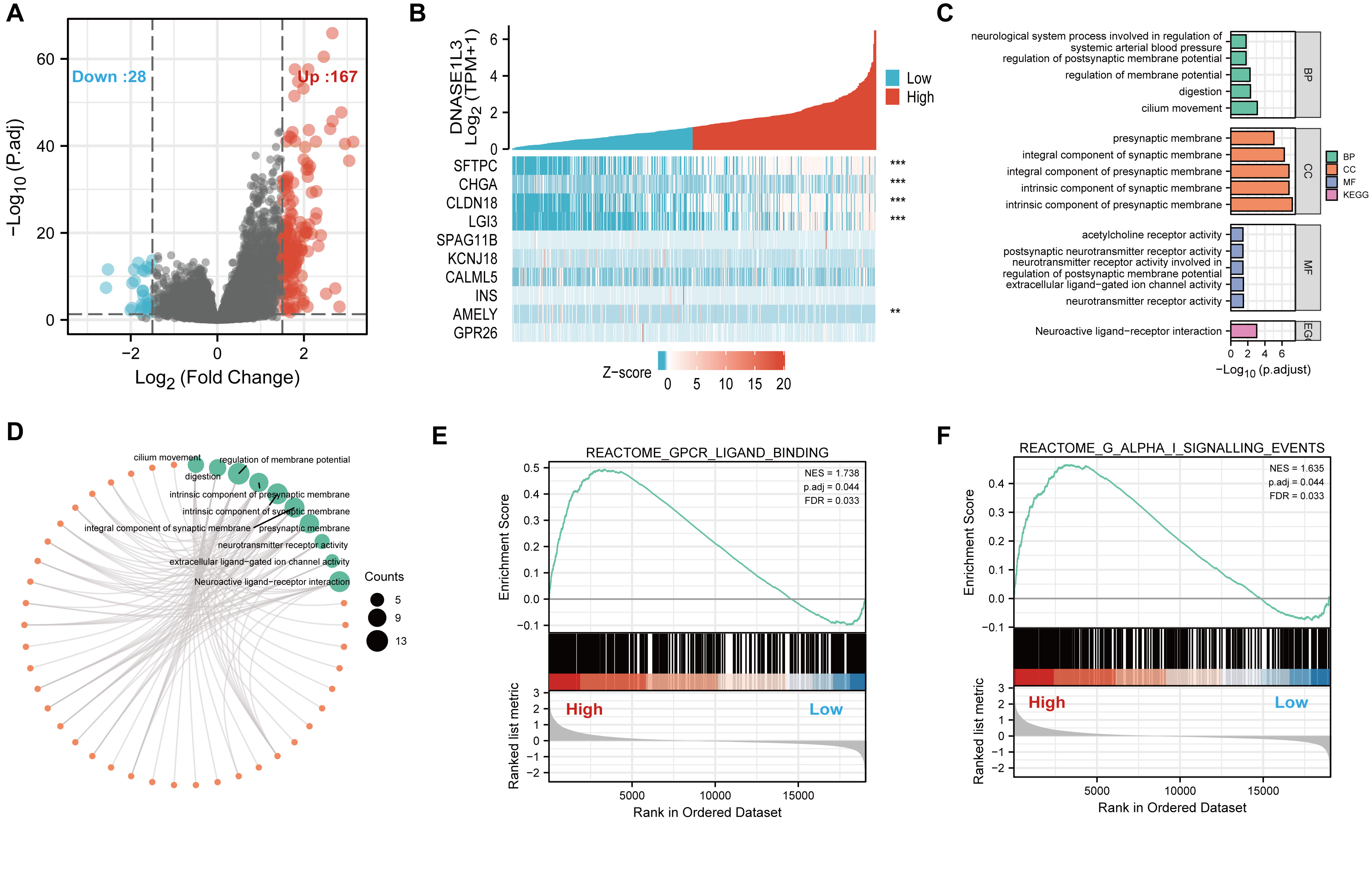

Supplement: Supplementary file 1 [file Image2.jpg]

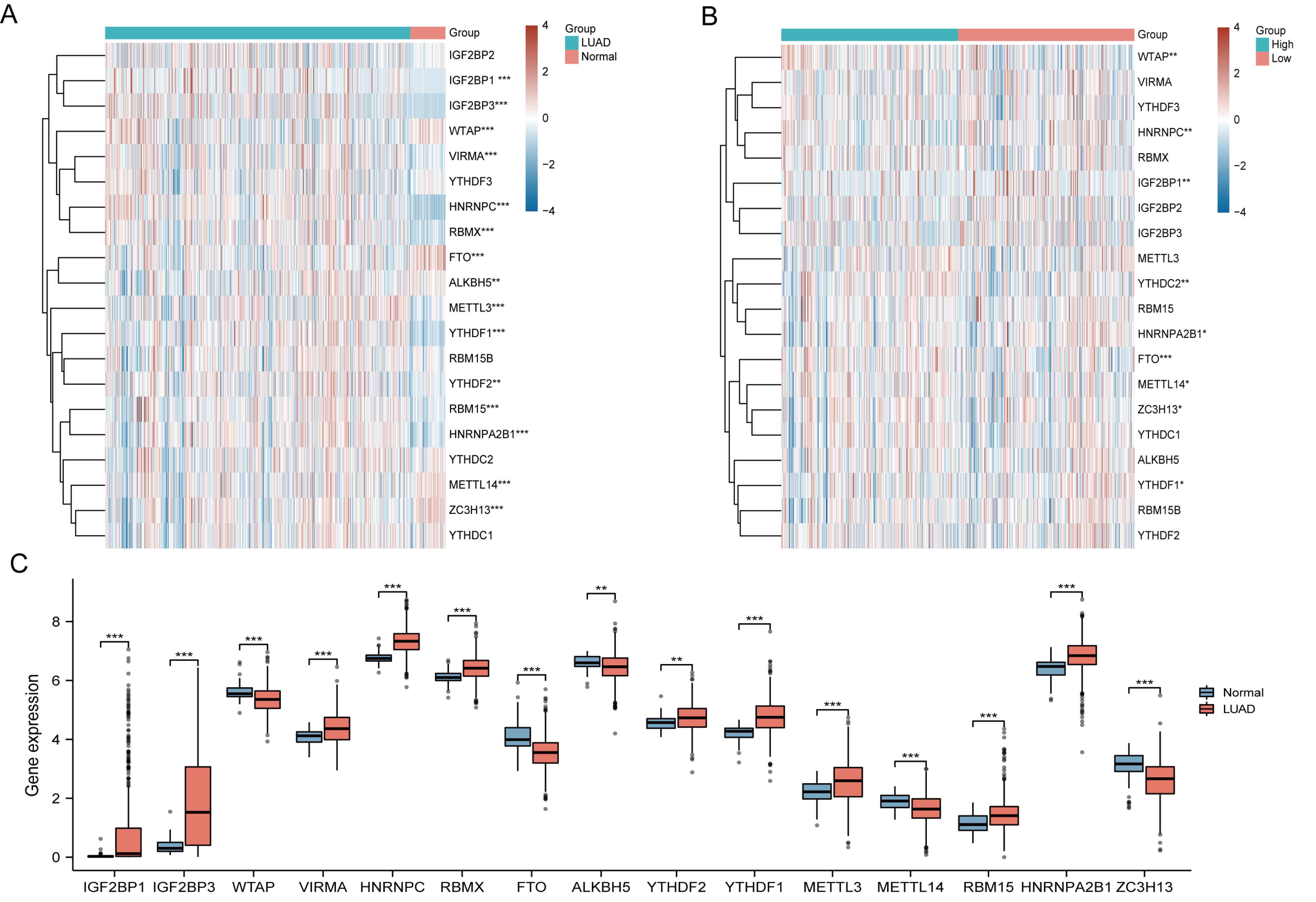

Supplement: Supplementary file 2 [file Image1.jpg]
